# Automated phenotyping for early vigour of field pea seedlings in controlled environment by colour imaging technology

**DOI:** 10.1371/journal.pone.0207788

**Published:** 2018-11-19

**Authors:** Giao N. Nguyen, Sally L. Norton, Garry M. Rosewarne, Laura E. James, Anthony T. Slater

**Affiliations:** 1 Australian Grains Genebank, Agriculture Victoria, Grains Innovation Park, Horsham, Victoria, Australia; 2 Agriculture Victoria, Grains Innovation Park, Horsham, Victoria, Australia; 3 Agriculture Victoria, AgriBio, Bundoora, Victoria, Australia; Università Politecnica delle Marche, ITALY

## Abstract

Early vigour of seedlings is a beneficial trait of field pea (*Pisum sativum* L.) that contributes to weed control, water use efficiency and is likely to contribute to yield under certain environments. Although breeding is considered the most effective approach to improve early vigour of field pea, the absence of a robust and high-throughput phenotyping tool to dissect this complex trait is currently a major obstacle of genetic improvement programs to address this issue. To develop this tool, separate trials on 44 genetically diverse field pea genotypes were conducted in the automated plant phenotyping platform of Plant Phenomics Victoria, Horsham and in the field, respectively. High correlation between estimated plant parameters derived from the automated phenotyping platform and important early vigour traits such as shoot biomass, leaf area and plant height indicated that the derived plant parameters can be used to predict vigour traits in field pea seedlings. Plant growth analysis demonstrated that the “broken-stick” model fitted well with the growth pattern of all field pea genotypes and can be used to determine the linear growth phase. Further analysis suggested that the estimated plant parameters collected at the linear growth phase can effectively differentiate early vigour across field pea genotypes. High correlation between normalised difference vegetation indices captured from the field trial and estimated shoot biomass and top-view area confirmed the consistent performance of early vigour field pea genotypes under controlled and field environments. Overall, our results demonstrated that this robust screening tool is highly applicable and will enable breeding programs to rapidly identify early vigour traits and utilise germplasm to contribute to the genetic improvement of field peas.

## Introduction

Field pea (*Pisum sativum* L.) is a legume crop that is widely grown around the world with annual production of c. 11 million metric tonnes produced from 6.9 million hectares of cultivated land [1]. Australia is among the 10 largest field pea producing countries, where the crop accounts for 20% of pulse production in South Australia and Victoria, and is the second largest pulse crop grown in Western Australia and third in New South Wales [2]. The Australian field pea industry produces approximately 400,000 metric tonnes of grain annually. Of this, about 191,000 metric tonnes are exported with the market value of $A80 million [3]. Field pea production brings profit for growers as a cash crop and provides other benefits for the farming systems such as non-cereal crop rotation and biological nitrogen fixation. Field pea and other crop legumes annually contribute approximately 5–7 million metric tonnes of biologically fixed nitrogen to cultivated soil, saving farmers $US 8–12 billion on nitrogen fertilizer costs globally [4, 5].

Like many other agricultural crops, field pea production is critically affected by biotic and abiotic stresses such as weeds, drought and heat [6]. Competition from weeds is one of the major biotic constraints affecting field pea production, which can result in yield loss up to 25% [7]. Field pea is a very poor competitor against weeds compared to other crops due to its weak early vigour at the seedling stage [8, 9]. Globally, herbicides are widely used for weed control during pea cultivation, and although instantly effective, overuse of herbicides with similar active ingredients and modes of action is resulting in herbicide resistant weed biotypes [10] and increased production costs [11]. Herbicides can also potentially affect rhizobium and symbiotic nitrogen fixation with field pea, causing smaller positive impacts on subsequent crop rotations [12]. Likewise, drought and heat stresses cause particularly greater yield losses in field pea crops [13, 14]. These abiotic stresses can have critical effects if they occur during flowering and grain filling by affecting reproductive organs and pod setting, thus reducing seed number [15, 16].

Previous studies suggest that breeding for tolerant varieties is one of the most effective strategies to cope with biotic and abiotic stresses, and early vigour traits have been considered an important selection criterion by field pea breeders [6, 17–19]. Although early vigour can be improved by using higher sowing rates and applying more nitrogen fertilizer, studies suggest that enhancing early vigour by genetic improvement is more effective and reliable [20]. Early vigour is the plant’s ability to establish quickly after sowing at the seedling stage and has been studied extensively in rice, wheat and other cereals [21–23]. Genetic studies in wheat showed that seedlings of vigorous genotypes can produce biomass rapidly, tiller earlier, have more leaves and have greater water and nitrogen use efficiency [24–26].

Although weed control can be managed by herbicides and other agronomical practices such as planting density, row spacing and orientation, use of vigorous genotypes with greater competitiveness is the most effective, non-chemical and environmentally friendly strategy [18, 27, 28]. Early vigour is also an important breeding trait for higher water use efficiency, especially in Mediterranean environments, since it minimizes soil water evaporation by boosting early vegetative ground cover [17, 29]. In water-limited environments, wheat genotypes with early vigour decreased water evaporation from the soil surface by reducing water loss by 90–110 mm and increased transpiration efficiency by 10% [30]. As a result, genotypes with early vigour have greater carbohydrate reserves before anthesis that can compensate for a photosynthesis reduction of up to 36% if drought occurs during the grain fill stage [31]. Early vigour is also an ideal trait of tropical crops grown in cold environments because it confers chilling tolerance [32]. Seedlings with high early vigour have a higher nitrogen uptake and photosynthetic nitrogen use efficiency [26, 33, 34]. Moreover, early vigour field pea varieties showed broader adaptation and yield maintenance under unfavorable growth conditions [35–37]. Thus, there is a pressing need to develop early vigour field pea genotypes via breeding in response to biotic and abiotic stresses.

Early vigour is a polygenic trait that requires a large volume of high quality phenotypic data to dissect its genetic composition into smaller manageable and measurable components [38]. Conventionally, early vigour trait assessment involves manual methods such as visual scoring, and measuring leaf area, plant height, and shoot biomass [18, 39]. Although attainable, these methods are labor intensive, subjective and prone to human errors, and are not suitable for large scale trials. Therefore, robust and high-throughput phenotyping tools and platforms that can generate reliable and high quality phenotypic data for genomic selection have become the rate-limiting step in field pea’s genetic improvement [40].

Non-destructive phenotyping technology using sensors and cameras can offer high-throughput and reproducible screens of large scale trials as well as reliable, high quality data and dynamic growth analysis of crops [41]. This technology has also been recommended for studying early vigour for nitrogen use efficiency in agricultural crops [22]. The technology was built to detect and quantify the spectral reflectances resulting from the interaction between plant parts and electromagnetic radiation at different spectral regions such as visible (VIS, 400–700 nm), near infrared (NIR, 700–1000 nm) and short-wave infrared (SWIR, 1000–2500 nm) [41, 42]. Software and computer vision enable the analysis of these reflectances to derive digital plant objects that can be used as surrogates for plant architectural morphology, biomass, and grain yield [43]. Several automated plant phenotyping platforms that comprise of growth facilities, sensors and cameras are commercially available and have been successfully applied in crop research under controlled and field conditions [22, 44–47]. For example, an automated high throughput phenotyping platform, PlantScreen (Photon Systems Instruments, Brno, Czech Republic) was used to assess the cold tolerance of field pea using digital colour imaging technology under controlled environments [48]. Similarly, Roth *et al*. [49] applied an aerial-based imaging phenotyping platform to estimate field pea biomass under field conditions. Vegetation indices such as normalized difference vegetation index (NDVI) derived from optical sensors has been used to analyse the growth of field pea and other crops under various field conditions [50, 51].

Here we report on the development of a high-throughput phenotyping method to evaluate early vigour of field pea in a controlled environment using an automated colour imaging technology and a comparative performance of the same genotypes under field conditions. The role of early seedling vigour in field pea breeding and production is also discussed.

## Materials and methods

### Plant material and experimental design

Forty-four genetically diverse field pea genotypes were used in these experiments to investigate early seedling vigour traits (S1 Table) [52]. Field pea seeds were carefully selected to ensure that seeds of the same genotype had similar size and shape to guarantee a similar level of germination. In the first experiment, field pea plants were grown in the greenhouse of Plant Phenomics Victoria, Horsham. Euro white pots (200 mm diameter x 190 mm deep, Garden City Plastics, Victoria, Australia) were filled by weight with 3.5 liters of potting mix consisting of 1,000 L legume mix (Biogro, SA), 1 kg Floranid 32, 1 kg Blue Macracote Coloniser Plus, 1 kg Nutricote N16, 1 kg Microplus trace element fertilizers, 225 g LibFer SP, 2 kg SaturAid, and 25 kg Lime. The pots were watered prior to sowing and placed on white saucers throughout the experiment to avoid water leaking on to the system. Three seeds were sown per pot and these were kept on rolling benches in the greenhouse of Plant Phenomics Victoria, Horsham. Each pot was thinned to one plant after seeds had germinated, approximately seven days after sowing (DAS), and blue wire cages were inserted into the pots to support plant growth. The colored cages facilitated differentiation of plant material from the support structure for imaging.

The first set of 352 plants (8 replicates per 44 genotypes) were loaded onto the fully automated plant phenotyping system of Plant Phenomics Victoria, Horsham, ten DAS and arranged in a randomised complete block design (RCBD). The automated plant phenotyping system is housed in a climate-controlled greenhouse and consists of conveyor belts, watering and weighing stations, and an imaging chamber with a Scannalyzer 3D imaging system (LemnaTec GmbH). A second set of 132 plants (44 genotypes, 3 replicates each) were grown on rolling benches for destructive harvest. The growth conditions in the greenhouse were controlled to 24°C during the day and 18°C during the night with a 12 h photoperiod. Enough water was applied automatically to maintain healthy plant growth during the experimental period and recorded into the system’s database (LemnaBase, LemnaTec GmbH).

In a second field experiment, all 44 pea genotypes were trialed in a RCBD design with three replicates during the 2016 winter–spring cropping season at the Plant Breeding Centre of Agriculture Victoria in Horsham, Victoria, Australia (36.74^o^S, 142.103^o^E; 133 m altitude). The experimental site has Vertosol heavy clay soil characteristics and a temperate climate with medium average annual rainfall of 450 mm [51]. Seeds were machine sown in plots (1 m width x 5 m length) at a density of 60 plants m^-2^. Fertilizer application and crop management for weed, pest and disease control were carried out in accordance with the standard practices in the area.

### Image capture and processing

**Fig 1:**
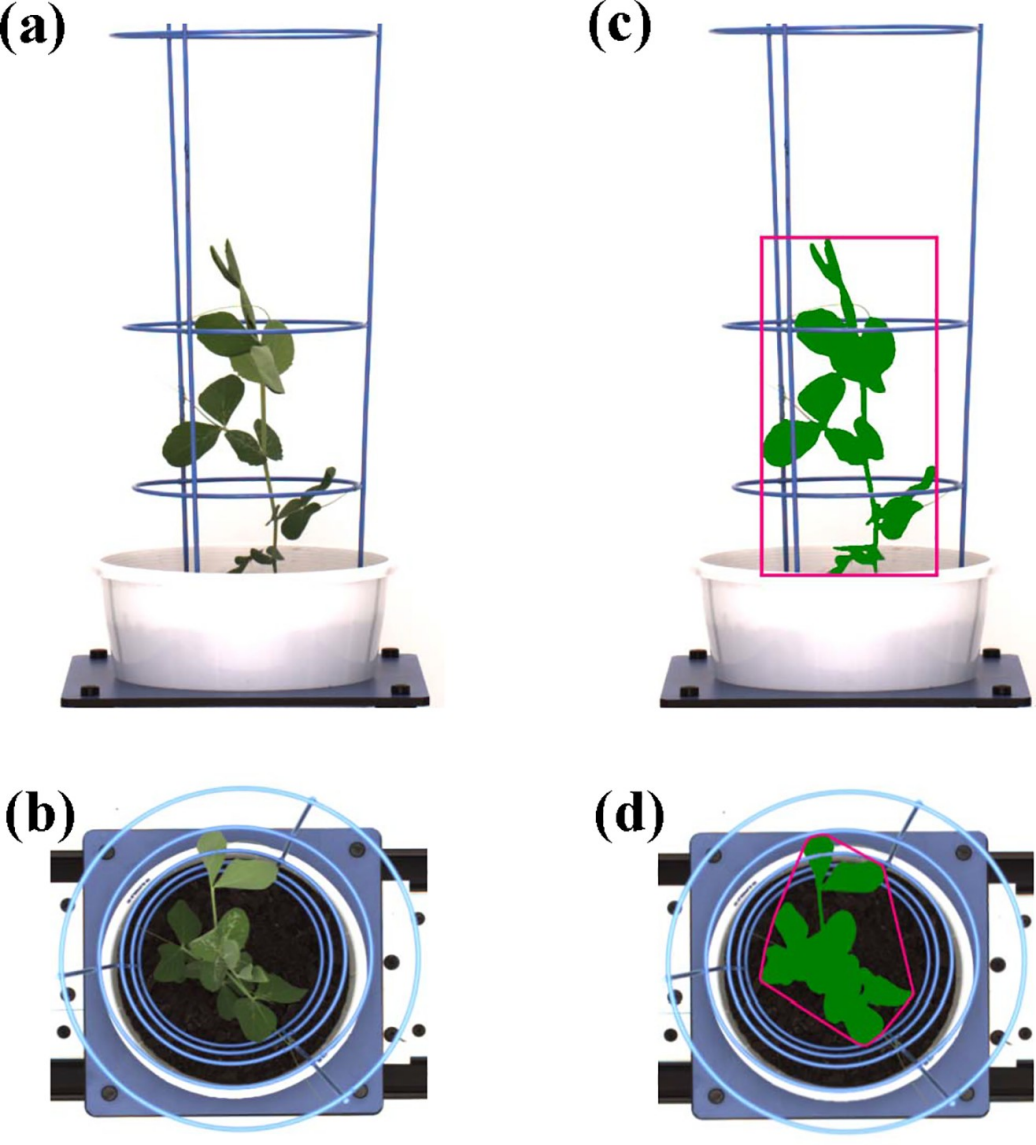
Image acquisition and analysis by the Scannalyzer 3D, plant-to-sensor imaging system. (a) and (b) are raw images of a side-view and top-view (pea cv. Alma); (c) and (d) processed images showing the identification of the corresponding side-view and top-view objects (bright green plant). Estimated shoot biomass is the pixel sum of highlighted green objects in processed images (c, d). The height of the pink rectangle in (c) is the plant height. The perimeter enveloped by the pink line in (d) is the top-view convex hull.

After loading onto the automated phenotyping system, plants were imaged daily by the Scannalyzer 3D plant-to-sensor imaging system which consists of two 28.8 megapixel red–green–blue (RGB) cameras (a side and a top camera), model Prosilica GT6600C (Allied Vision Technologies, Stadtroda, Germany). Side-view RGB images were acquired from three sides of the plant after consecutive rotations of 0, 120 and 240 degrees, and a top-view RGB image was taken from above the plant (Fig 1A and 1B). Captured images were automatically recorded in LemnaBase and analyzed by LemnaGrid software (LemnaTec GmbH).

The region of interest consisting of the whole plant in raw images was separated from the background by LemnaGrid. In the subsequent steps, the image noise was removed from the region of interest and clear digital plant objects were determined (Fig 1C and 1D). The pixel sums of digital plant objects were generated by LemnaMiner software (LemnaTec GmbH) and subsequently used to estimate several morphological and physiological features of the plants (Table 1).

**Table 1:** List of field pea traits measured using digital colour imaging and conventional destructive methods.

Traits	Abbreviation	Unit	Description
Estimated shoot biomass	EB	kilopixel (kPix)	Estimated biomass accumulation of plants calculated from pixel sums of three side-view and top-view images of the plant
Top-view area	TVA	kPix	Estimated pixel sums of the top-view image
Top-view convex hull	TVCH	kPix	The smallest perimeter enveloping the top-view image of the plant
Top-view compactness	TVCOM	Generic	The ratio of leaf area per top-view convex hull area
Estimated plant height	EH	Pixel (Pix)	Estimated maximum distance from bottom to top of plant
Estimated water use efficiency	eWUE	kPix.kgwater^-1^	The ratio of estimated biomass per total amount of supplied water
Relative growth rate	RGR	kPix.day^-1^	RGR = ($\bar{\ln\left(W2\right)}‑\bar{\ln\left(W1\right)}$)/(t_2_-t_1_), where $\bar{\ln\left(W1\right)}$ and $\bar{\ln\left(W2\right)}$ are means of ln-transformed estimated shoot biomass at days t_1_ and t_2_ [53]
Measured plant height	MH	cm	Maximum distance from the cut end to the tip of the main stalk
Measured shoot biomass	MB	Gram (g)	Destructive biomass harvest at 25 and 39 DAS
Measured leaf area	LA	cm^2^	Total leaf area per plant per pot
Measured water use efficiency	mWUE	g.kg water^-1^	The ratio of measured shoot biomass per total amount of supplied water

### Manually destructive harvest

The second set of 132 field pea plants were destructively harvested at 25 DAS after being loaded onto the automated plant phenotyping platform and imaged the night before. Whole plants were weighed using a UniBloc electronic balance (Shimadzu, Melbourne, Australia) to determine fresh shoot biomass per pot (Table 1). The plant height of single plants was determined by measuring from the cut end from immediately above the soil to the tip of the main stalk (Table 1). All leaves from single plants were detached from stalks and leaf area was measured by a Portable Area Meter, model LI-3050A (LI-COR Inc., Lincoln, Nebraska, USA) (Table 1). The remaining 352 field pea plants were unloaded from the automated plant phenotyping platform and destructively harvested at 39 DAS. Fresh shoot biomass was determined as described above.

### Normalized difference vegetation index (NDVI) measurements

Early vigour of pea genotypes grown in the field in the second experiment was assessed by a crop growth index NDVI derived from spectral reflectance measured by the Crop Circle sensing equipment (ACS-470; Holland Scientific Inc., Lincoln, NE, USA). NDVI was calculated using the formula from Rouse *et al*. [54]; (R760 –R670)/(R760+R670), where R670 and R760 are reflectance (R) at 670 nm (VIS region) and 760 nm (NIR region), respectively. Spectral reflectance signals were captured by scanning Crop Circle horizontally 0.75–0.90 m over the plant canopy at 52 DAS as described by Nguyen *et al*. [51].

### Plant growth model and statistical analyses

Since biomass accumulation of cereal crops generally follows a nonlinear growth pattern [55], the “broken-stick” statistical model fitting two straight lines using regression split-line function of GENSTAT statistical software version 18.0 (VSN International Ltd, Hemel Hempstead, UK) was used to identify the linear growth phase of field pea plants as described by Kong *et al*. [56] and Kholová *et al*. [57].

Imaging-derived and manually measured data were checked for outliers by using boxplot function of GENSTAT statistical software and presented as means of eight replicates per genotype, with exception to the plants destructively harvested at 25 DAS as this data was a mean of three replicates. One-way analysis of variance (ANOVA) was performed to determine any varietal effects and linear regressions and Pearson’s correlation coefficients (r) were used to determine the relationship between estimated and measured plant traits by using R statistical software (version R-3.5.0) [58].

## Results

### Validation of nondestructive imaging phenotyping of growth indices

**Fig 2:**
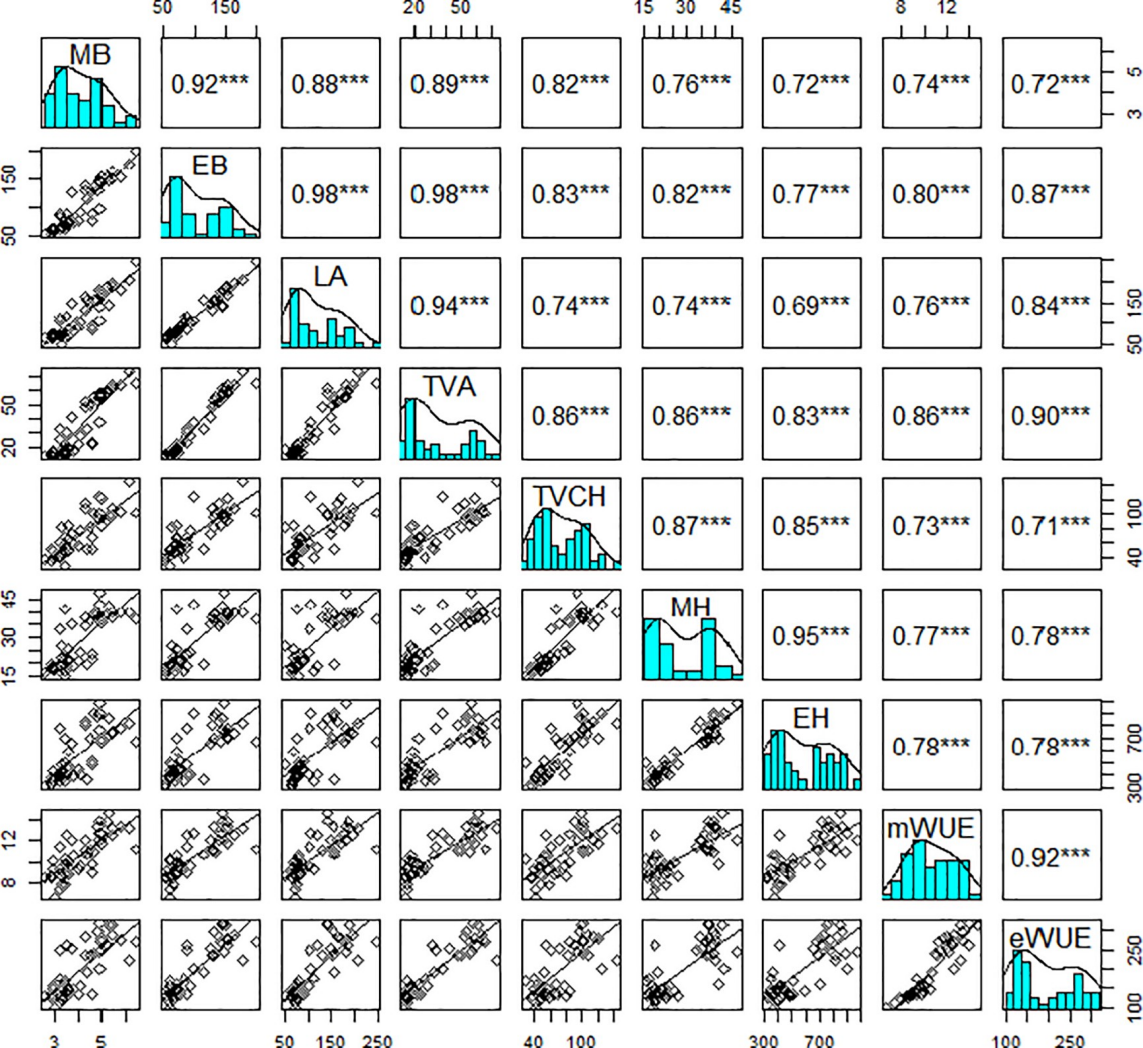
Relationship between estimated with measured morphological and physiological parameters of pea genotypes. The cyan panels are the histograms of individual traits. Panels above and below the diagonal of each cyan panel are Pearson’s correlation coefficients (r) and bivariate scatter plots with trend lines, respectively. The asterisks indicate the statistically significant level (*** p<0.001). MB, measured shoot biomass; EB, estimated shoot biomass; LA, measured leaf area; TVA, top-view area; TVCH, top-view convex hull; MH, measured plant height; EH, estimated plant height; mWUE, measured water use efficiency; eWUE, estimated water use efficiency; sample number = 44.

To validate the suitability of image analysis to predict the early vigour phenotype of field pea under controlled environments, we first analysed the estimated values captured through imaging against the measured values from destructive analysis of morphological and physiological parameters of 44 field pea genotypes (Table 1; Fig 1). The results showed that the estimated and measured traits are highly correlated for all 44 field pea genotypes (Fig 2). The most important estimated trait, estimated biomass (EB) is strongly correlated with measured traits such as measured biomass (MB) and leaf area (LA) with high Pearson’s correlation coefficients (r = 0.92 and 0.98, respectively; Fig 2). Similarly, two estimated traits top-view area (TVA) and top-view convex hull (TVCH) were also highly correlated with LA (r = 0.94 and 0.74; Fig 2). Other estimated traits such as estimated height (EH) and estimated water use efficiency (eWUE) also show high correlation with the corresponding measured traits (r = 0.95 and 0.92, respectively; Fig 2). Overall, these estimated and manually measured morphological and physiological parameters are highly correlated.

### Dynamic growth analysis of field pea genotypes

**Fig 3:**
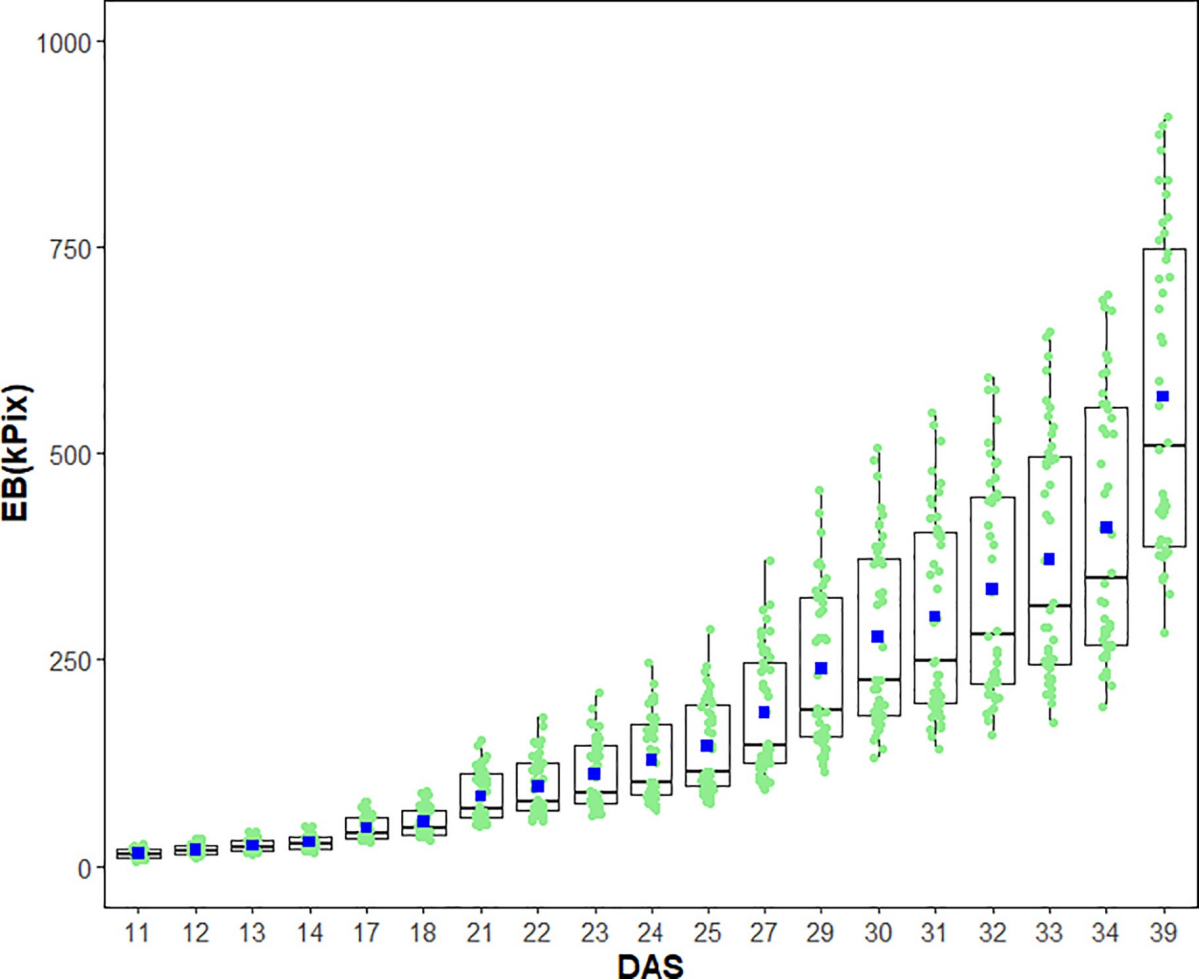
Dynamic growth of field pea genotypes. Boxplots of the estimated shoot biomass accumulation over the growth period for 44 field pea genotypes. EB, estimated shoot biomass; DAS, days after sowing. In each box, the black line is the median; the blue squares are the mean; the light green dots are EB of individual field pea genotypes.

Since early seedling vigour is strongly influenced by shoot biomass accumulation during the linear growth phase, we determined the earliest time point where estimated early vigour can be used to compare the performance of all field pea genotypes. Unlike conventionally destructive sampling methods, nondestructive digital imaging allows the calculation and observation of dynamic growth and shoot biomass accumulation of plants over time. Our data showed that the mean EB increased over the period from 11 to 39 DAS (Fig 3).

These boxplots showed that the EB of 44 field pea genotypes could be separated into two distinct stages; the lag and the linear phases (Fig 3). Using the broken-stick statistical model, we identified the coordinates X which is the reference point of the days after sowing and Y, the estimated biomass at X of the breakpoints that separate the two growth phases for each genotype of field pea (Table 2). Data showed that the broken-stick model fitted well with the growth of all field pea varieties as indicated by the high adjusted coefficients of determination (R^2^ >0.99; Table 2). The slope of the regression after the breakpoint (slope 2) of all varieties exceeds that before the breakpoint (slope 1).

**Table 2:** Regression parameters as determined by the split-line linear regression model of 44 field pea genotypes^a^.

N^o^	Variety	Coordinate X (day)^b^	Coordinate Y (kPix)	Slope 1	Slope 2
1	Alma	20.40	104.50	9.18	42.16
2	Bluey	26.11	89.69	5.71	23.03
3	Bohatyr	24.48	166.20	12.06	40.04
4	Bonzer	25.79	93.86	6.10	21.44
5	Bundi	25.77	90.49	5.83	19.20
6	Collegian	25.52	209.20	13.65	47.16
7	Cooke	25.65	189.00	12.84	56.16
8	Cressy Blue	23.42	160.50	12.60	49.27
9	Derrimut	23.50	159.50	12.31	48.08
10	Dinkum	25.83	108.43	7.32	27.95
11	Dunn	22.40	138.40	11.30	32.93
12	Dundale	24.43	156.80	11.15	39.22
13	Dunwa	23.52	124.82	8.95	30.77
14	Excell	25.81	95.75	6.16	23.26
15	Glenroy	25.63	161.81	10.48	34.41
16	Helena	25.53	145.73	10.05	40.03
17	Jupiter	24.41	113.94	7.92	29.52
18	Kaspa	25.61	91.73	5.86	20.26
19	Kiley	25.64	97.58	6.36	19.34
20	King	26.22	107.66	6.65	30.91
21	Laura	25.63	165.30	11.37	45.51
22	Magnet	25.56	93.93	6.32	23.98
23	Maitland	25.80	137.93	8.62	31.56
24	Maki	26.01	84.65	5.33	20.33
25	Moonlight	25.59	97.33	6.27	21.78
26	Morgan	25.88	110.42	6.90	24.94
27	Mukta	26.26	78.10	4.65	17.46
28	Parafield	23.53	136.10	10.05	35.95
29	Paravic	25.98	93.54	6.12	22.40
30	PBA Gunyah	25.56	96.06	6.20	20.00
31	PBA Oura	25.87	77.13	4.79	18.05
32	PBA Pearl	26.03	84.67	5.42	20.11
33	PBA Percy	25.71	175.42	11.42	42.23
34	PBA Twilight	26.14	83.16	5.05	18.12
35	PBA Wharton	25.51	87.60	5.55	18.73
36	Santi	25.97	81.05	5.08	18.78
37	Snowpeak	25.58	101.93	6.95	25.09
38	Soupa	25.65	187.80	12.61	48.05
39	Sturt	26.16	153.58	9.66	45.74
40	SW Celine	25.95	93.74	6.07	23.42
41	Whero	22.59	162.20	12.70	34.60
42	White Brunswick	25.68	193.67	12.77	49.75
43	Wirrega	25.94	177.10	11.67	49.85
44	Yarrum	26.15	75.42	4.82	14.44

^a^Adjusted R^2^ > 99%

^b^Coordinate X is the reference point of the days after sowing and Y is the estimated biomass at X, where the linear regression was split or “broken”; slope 1, the coefficient of the regression before breakpoint; slope 2, the coefficient of the regression after the breakpoint.

**Fig 4:**
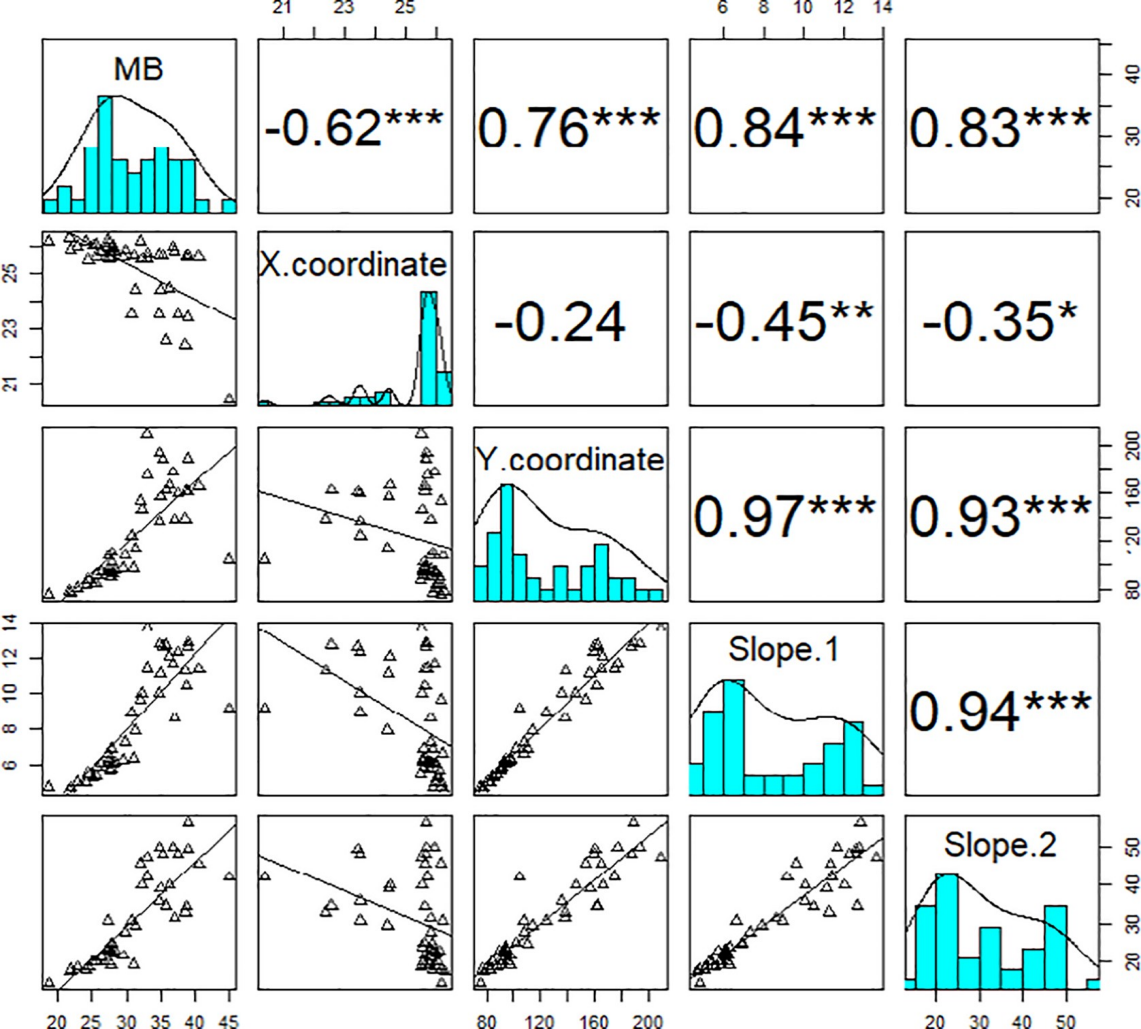
Relationship between early vigour of field pea genotypes and parameters of the “broken-stick” model. The cyan panels are the histograms of measured biomass and parameters of the broken-stick model. Panels above and below the diagonal of each cyan panel are Pearson’s correlation coefficients and bivariate scatter plots with trend lines, respectively. The asterisks indicate the statistically significant level (*** p<0.001). MB, measured shoot biomass; X and Y coordinates, the reference points of the days after sowing and the estimated biomass at X; Slope 1, the coefficient before the break point; Slope 2, the coefficient after the break point; sample number = 44.

Pearson’s correlation analysis between MB, and parameters of the broken-stick model showed that MB was highly negatively correlated with X coordinate, while it was highly positively correlated with Y coordinate, slope 1 and slope 2 (Fig 4).

Data also showed that X coordinates of several varieties were between 21–23 DAS; such as Alma, Dunn, and Whero, whereas many other varieties had their X coordinates at later dates over 26 DAS; Bluey, King, Maki, Mukta, PBA Pearl, PBA Twilight, Sturt, and Yarrum (Table 2). The latest X coordinates of several field pea genotypes was approximately 26.3 DAS, suggesting that any growth points after this date fell into linear growth phase and only EB values from this point forward should be used for the comparison of early vigour across 44 field pea varieties (Table 2). For consistency, we used the estimated morphological and physiological values collected at 27 DAS hereafter to compare the performance of field pea genotypes in the following sections.

### Assessment of early vigour traits of field pea genotypes

To determine how well the estimated traits correlate with early seedling vigour of field pea, we compared the MB harvested at 39 DAS, a time point lying in the linear growth phase, against estimated morphological and physiological values of 44 pea genotypes at 27 DAS (Table 3). Overall, the performance of all varieties estimated morphological and physiological values per genotype were relatively consistent with MB (Table 3, Fig 2). However, there was significant variation among estimated traits, with the most consistent traits relative to MB being EB, TVA, eWUE and to a lesser extent for TVCH, EH, and RGR, while TVCOM was the least consistent trait (Table 3, Fig 2). For example, varieties Alma, Laura, Cressy Blue, and Cooke are the most vigorous genotypes, whereas, Santi, PBA Oura, Mukta, and Yarrum are the least vigorous genotypes in respect of MB (Table 3). However, Laura has a significantly smaller EB, TVA, EH, and eWUE compared to Cressy Blue.

**Table 3:** Comparative performance of pea genotypes for early vigour^a^.

Ranking	Variety	Leaf type	MB	EB	TVA	TVCH	TVCOM	EH	RGR	eWUE
1	Alma	C	44.96	370.40	142.61	298.39	0.49	1148.75	0.13	189.34
2	Laura	C	40.65	237.32	92.13	175.10	0.53	876.25	0.14	122.27
3	Cressy Blue	C	39.04	317.51	121.13	257.44	0.48	1231.50	0.15	165.59
4	Cooke	C	39.00	277.25	91.61	163.89	0.58	1043.50	0.14	146.98
5	Glenroy	SL	38.76	216.88	75.71	251.45	0.32	1088.25	0.12	112.96
6	Dunn	C	38.69	283.58	112.49	259.72	0.46	991.38	0.15	152.33
7	Derrimut	C	37.50	310.81	105.97	202.29	0.56	1004.13	0.14	163.21
8	Maitland	SL	37.04	186.55	67.25	253.68	0.28	1165.63	0.13	96.51
9	Wirrega	C	36.79	241.75	82.10	150.91	0.55	988.88	0.13	123.26
10	Bohatyr	C	36.36	259.45	80.93	144.81	0.56	651.75	0.12	134.19
11	Whero	C	35.90	298.66	119.36	233.38	0.52	1116.00	0.12	151.24
12	Soupa	C	35.35	261.15	81.58	144.28	0.58	898.75	0.12	133.25
13	Dundale	C	35.01	251.54	92.98	169.53	0.55	1019.50	0.12	131.90
14	White Brunswick	C	34.90	268.41	84.34	155.34	0.55	1074.00	0.12	138.85
15	Parafield	C	34.85	245.31	85.38	199.62	0.48	813.88	0.11	127.77
16	PBA Percy	C	33.09	234.82	83.56	175.92	0.51	994.38	0.12	124.48
17	Collegian	C	32.99	284.99	91.29	162.04	0.57	980.25	0.11	146.14
18	Helena	C	32.25	211.37	77.33	138.72	0.56	879.63	0.13	108.89
19	Sturt	C	32.05	205.84	74.67	143.46	0.53	992.63	0.13	105.41
20	Jupiter	C	31.39	187.12	62.48	123.49	0.52	632.63	0.13	95.05
21	Kiley	SL	31.09	145.33	32.39	134.62	0.25	522.63	0.11	64.84
22	Dunwa	C	30.79	221.54	83.97	171.84	0.50	1014.63	0.12	118.38
23	Dinkum	SL	29.94	148.44	31.35	128.18	0.30	585.50	0.13	74.21
24	Moonlight	SL	29.54	133.41	29.48	178.40	0.18	757.25	0.13	67.99
25	Excell	SL	28.40	128.68	28.60	85.82	0.34	560.25	0.12	68.31
26	Paravic	SL	28.13	123.24	23.49	81.98	0.30	577.50	0.13	62.17
27	Snowpeak	SL	27.86	141.92	30.81	146.91	0.22	674.13	0.13	73.96
28	Morgan	SL	27.80	145.70	44.21	171.78	0.26	1126.13	0.12	77.97
29	Bundi	SL	27.74	118.50	25.86	93.52	0.30	608.75	0.12	62.50
30	Bluey	SL	27.64	117.92	23.97	80.96	0.34	449.86	0.12	59.24
31	Magnet	SL	27.55	132.57	33.83	139.77	0.24	572.13	0.14	67.39
32	PBA Gunyah	SL	27.51	128.43	28.34	117.36	0.26	609.00	0.12	65.84
33	King	C	27.44	141.69	41.43	71.16	0.60	456.50	0.11	72.28
34	SW Celine	SL	27.14	125.54	25.23	104.04	0.24	591.13	0.12	67.20
35	Bonzer	SL	26.38	125.79	30.60	105.32	0.32	601.63	0.12	64.12
36	Kaspa	SL	25.75	123.93	27.93	134.95	0.22	605.50	0.13	63.18
37	PBA Pearl	SL	25.55	110.04	23.32	101.65	0.23	680.13	0.12	57.03
38	Maki	SL	25.14	110.17	23.81	72.28	0.38	502.38	0.11	56.12
39	PBA Wharton	SL	24.53	118.67	26.10	115.04	0.25	612.38	0.12	63.91
40	PBA Twilight	SL	24.19	103.62	22.29	96.21	0.24	569.25	0.11	55.87
41	Santi	SL	22.93	105.58	24.13	91.93	0.28	496.63	0.11	54.78
42	PBA Oura	SL	21.95	101.39	22.99	92.14	0.26	538.25	0.12	54.13
43	Mukta	SL	21.79	96.17	20.15	77.05	0.27	472.38	0.11	48.76
44	Yarrum	SL	18.64	92.02	18.01	59.48	0.31	429.00	0.11	49.30
***ANOVA***										
*p*		-	<0.001	<0.001	<0.001	<0.001	<0.001	<0.001	0.047	<0.001
s.e.d		-	2.36	20.2	7.6	25.4	0.038	58.56	0.011	10.34
LSD (*p* = 0.05)		-	4.65	39.8	15	50	0.076	115.24	0.022	20.34

^a^ Early vigour traits are represented by measured fresh biomass harvested at 39 DAS and estimated morphological and physiological values at 27 DAS.

Data are means (n = 8). In a column: dark green cells, the highest values; dark red cells, the lowest values; C, conventional; SL, semi-leafless; MB, measured shoot biomass (g); EB, estimated shoot biomass (kPix); TVA, estimated top-view area (kPix); TVCH, estimated top-view convex hull (kPix); TVCOM, estimated top-view compactness; EH, estimated plant height (Pix); RGR, relative growth rate (kPix.day^-1^); eWUE, estimated water use efficiency; s.e.d, standard error differences of the means.

As expected, conventionally leafed field pea genotypes had higher biomass accumulation and TVCOM than semi-leafless lines, indicating their stronger early vigour. Nevertheless, it’s noteworthy to mention that some semi-leafless varieties such as Glenroy and Maitland also had strong early vigour as indicated by high biomass accumulation (Table 3).

### Correlation between normalized difference vegetation index captured in the field with estimated shoot biomass and top-view area in controlled environment

**Fig 5:**
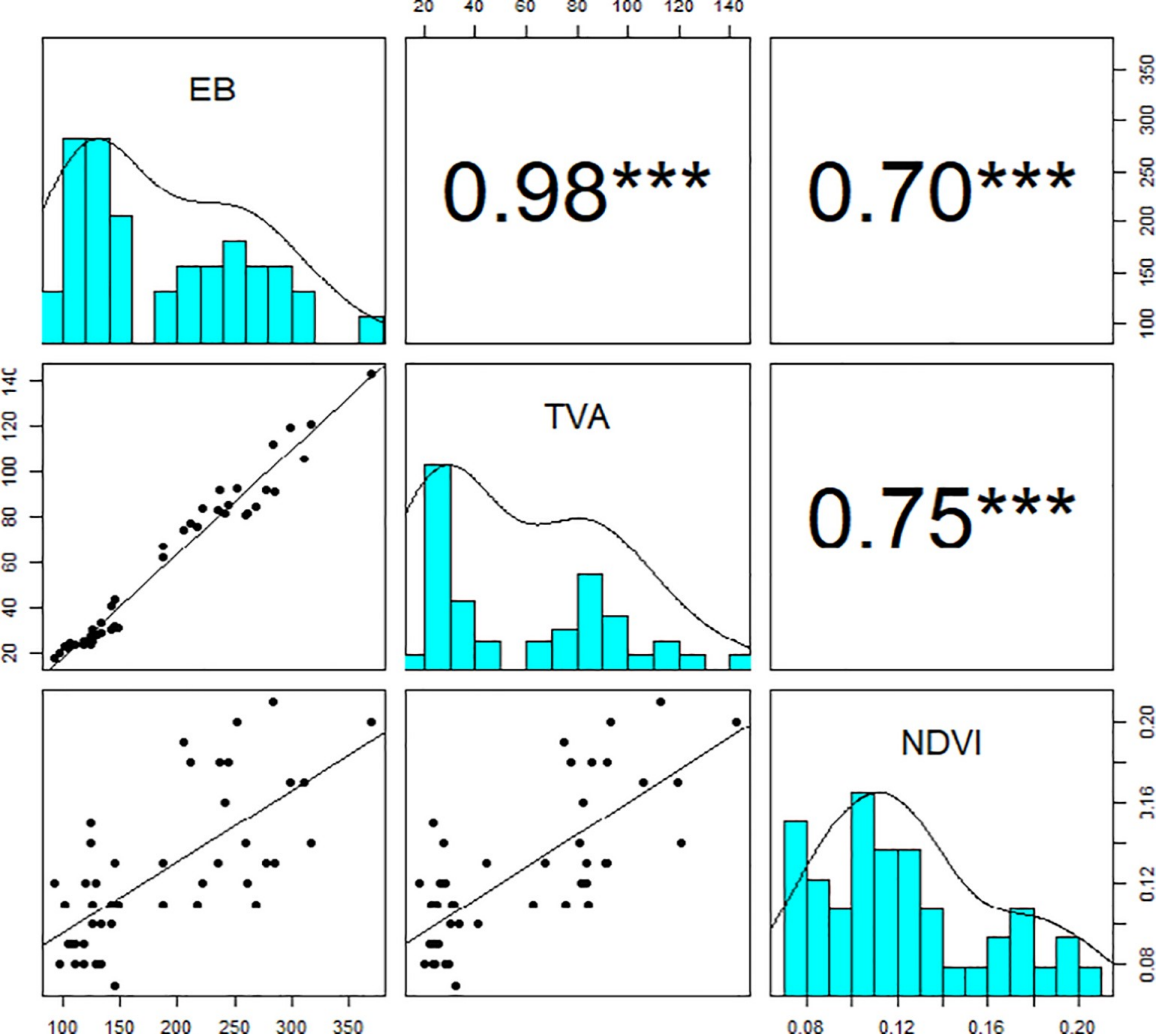
Comparative performance of 44 field pea genotypes under field and controlled environment. The cyan panels are the histograms of estimated biomass (EB) and top-view area (TVA) collected at 27 DAS in Plant Phenomics Victoria, Horsham and the vegetation indices (NDVI) measured by Crop Circle at 52 DAS in the field. Panels above and below the diagonal of each cyan panel are Pearson’s correlation coefficients and bivariate scatter plots with trend lines, respectively. The asterisks indicate the statistically significant level (*** p<0.001). Sample number = 44.

To compare the performance of pea genotypes in the field and controlled environment, we identified the association between NDVI values collected from the field trial and EB and TVA derived from imaging-based phenotyping in the Plant Phenomics Victoria, Horsham (Fig 5). Our data showed that NDVI values captured by Crop Circle are highly correlated with EB and TVA for all pea genotypes with correlation coefficients (r) of 0.7 and 0.75, respectively (Fig 5). This suggests that field pea genotypes selected for early vigour using this imaging method from the greenhouse are likely to show their early vigour under field conditions.

## Discussion

The overall aim of this research was to develop a high-throughput and reliable phenotyping method to rapidly assess early seedling vigour of field pea using digital colour imaging technology in a controlled environment. This is a crucial trait that contributes to biological weed control [27], drought tolerance and water use efficiency [17, 29, 30], and improved carbohydrate reserves [31] and nutrient uptake [26, 34, 59] in crops. Genetic improvement for early vigour has been proposed for many agricultural crops such as rice [38, 60], wheat [24, 61] and maize [62]. Therefore, developing new genotypes with improved early seedling vigour is a priority for field pea breeding programs [6, 17]. However, early seedling vigour is a complex trait [38] and the lack of a robust, high-throughput, and reliable phenotyping method that is powerful enough to dissect the component traits for genetic improvement is a bottleneck [63, 64]. The development and validation of high throughput phenotyping techniques, such as this one, will help to remove this limitation.

Seedling vigour can be broken down into several components including biomass accumulation, canopy coverage, and plant height. These components are generally evaluated visually and destructively [18, 35, 39]. The recent emergence of digital colour imaging techniques for plant phenotyping has provided opportunities to non-destructively evaluate morphological and physiological features of agricultural crops such as field pea, wheat, maize, barley and rice under various growing conditions [43, 48, 65–67]. To identify a robust and high-throughput plant phenotyping method applicable to screen field pea for early vigour traits, an experiment on genetically diverse field pea genotypes was conducted on the automated plant phenotyping platform of Plant Phenomics Victoria, Horsham over the winter–spring cropping season of 2016. The digital colour imaging data acquired by the plant phenotyping platform showed that estimated traits such as EB, TVA, EH, are highly correlated with shoot biomass accumulation, leaf area coverage and plant height. This confirms that they can be used as representations of these traits to predict the early vigour of field pea. These high correlations between digitally estimated and conventionally measured morphological and physiological traits were also observed by other studies using a similar automated imaging phenotyping system [55, 68]. The data also demonstrated that the early vigour across field pea genotypes can be quantitatively assessed as early as 27 DAS without destructive analysis of the plants, that can contribute to a cost-effective screen of field pea plants. Economical rapid assessment of less than 30 days will also allow for the rapid assessment of larger populations of accessions from genebanks for gene discovery, or even breeding populations for the development of varieties with a combination of desirable traits including early vigour.

Water use efficiency is one beneficial trait in field pea breeding for water-limited environments [19]. It can be defined as a unit of crop production gain per unit of water supply, and increased WUE is generally associated with higher grain yield [11]. The plant phenotyping platform used here can capture digital imaging data to assess early vigour traits and precisely record the amount of water supplied to each pot, which has also been reported by Ge *et al*. [69]. This is one advanced feature of an automated plant phenotyping method in comparison to conventional methods, where the former allows precise monitoring and documenting of the supplied water amount. Therefore, eWUE can be directly determined by the estimated biomass and known water usage for each field pea genotype. Armstrong *et al* [37] used a conventional phenotyping method to study WUE in field peas and discovered that conventionally leafed and vigorous varieties, such as Dundale and Wirrega, had higher WUEs than the most vigorous semi-leafless variety Dinkum, which is in line with findings from the current research. This again confirms the feasibility of the automated imaging phenotyping system for WUE studies. However, it’s important to note that estimated traits do not fully account for the variation observed in measured traits when compared to other imaging methods due to overlapping leaves that cannot be differentiated in the captured images (Fig 1). This limitation of the phenotyping method by imagery has been discussed extensively elsewhere [70]. Some estimated traits are more highly correlated with measured traits than others. These variations suggest that multiple estimated traits should be taken into consideration when comparing early vigour of field pea genotypes to ensure the selection of the best performers.

Most of the semi-leafless field pea genotypes have reduced internode length and this genetic combination is favoured by field pea breeding programs due to improved lodging tolerance and greater air flow through the canopy that results in reduced disease pressure [71]. However, the trait combination of semi-leafless and short internode length has a direct impact on early seedling vigour, leaf area index, and ground cover [35]. Interestingly, some semi-leafless varieties such as Glenroy and Maitland had very strong early seedling vigour. Thus, such early vigour semi-leafless genotypes identified in the present study might possess multiple desirable, inheritable attributes, and should be recommended for selection as parents in field pea breeding programs.

Although greenhouse screening may be a cost-effective method to select the best performers for breeding programs, the screening outcomes need to be further validated under field conditions, as crop performance is highly influenced by environmental conditions such as soil, water availability, and temperature [17, 72, 73]. In the present study, NDVI values as a proxy of early vigour collected from the field trial using Crop Circle were well associated with EB and TVA, confirming that selecting field pea genotypes using the automated imaging phenotyping method were reasonably reliable under field conditions. The stronger correlation between NDVI and TVA was probably due to both metrics being derived from 2D observations downward from sensors to the top of the plants and canopies, respectively; whereas, EB was calculated from 3D observations of plants. Digital cameras have been successfully applied to study early vigour traits and ground cover percentage of field pea and other crops [35, 67, 74]. Therefore, it is possible that similar sensory or imagery systems mounted on ground- or aerial-vehicles could be applied to render the high-throughput phenotyping of pea under field conditions [22, 44, 46, 49, 75–77] and this should be included in future investigations.

Generally, early seedling vigour directly contributes to crop establishment, biotic and abiotic stress tolerance and finally, grain yield at harvest [19]. However, no direct association between early seedling vigour with grain yield of crops grown under favourable conditions has been reported [24, 78]. In contrast, there is mounting evidence of a relationship between early seedling vigour and grain yield of field pea under adverse growing conditions such as limited rainfall environments [37]. Early seedling vigour of some crops including field pea is not a trait suitable for all environmental conditions, but it could be a valuable phenotypic trait for targeted environments [50]. In sub-tropical environments where crops rely on water availability stored in the soil profile, early seedling vigour might even be a harmful trait, since plants exhaust reserved water quickly resulting in terminal drought at the reproductive phase. In temperate and Mediterranean type climates such as southern Australia, cropping systems mainly depend on seasonal rainfall where early vigour enhances ground cover, minimizing water losses through run-off and evaporation, and facilitating crop growth [11, 14].

Armstrong and Pate [36] reported on the trialing of six contrasting morphological field pea genotypes at three different rainfall locations in Western Australia. Their results showed that Wirrega, a conventionally leafed and early vigorous variety, outperformed other varieties in the dry regions, which was attributed to better ground cover, long main stem, and larger green area index; whereas, its performance was much poorer than the others under cool and high rainfall environments [36]. In the present study, no grain yield data of the pea genotypes was collected, though it might be noteworthy to investigate the contribution of early vigor to grain yield in future studies, particularly under adverse growing conditions. Apparently, weak vigour genotypes are undesirable for any growth conditions and too vigorous genotypes are also not ideal for some environments. Therefore, genotypes with certain degrees of early vigour should be selected so that they can perform best in targeted environments.

## Conclusions

We have developed a high-throughput digital image phenotyping method to assess early seedling vigour of genetically diverse field pea genotypes using an automated plant phenotyping platform. Our results have demonstrated that the imaging method is fully capable of detecting variations of early vigour traits of field pea varieties under controlled environments, and this has been further validated for their comparative performance under field conditions. Therefore, this robust screening method is highly applicable and will enable breeding programs to rapidly identify early vigour traits and utilise germplasm to contribute to the genetic improvement of field peas. To our best knowledge, this is a very first method detailing the application of high-throughput automated imaging phenotyping technology to assess early vigour of field pea under a controlled environment.

## Supporting information

S1 Table Origin of 44 genetically diverse field pea varieties used in this study. (DOCX)
