# Correction: Automated phenotyping for early vigour of field pea seedlings in controlled environment by colour imaging technology

**DOI:** 10.1371/journal.pone.0208972

**Published:** 2018-12-06

**Authors:** The PLOS ONE Staff

There are errors in the Funding section. The correct funding information is as follows: This research was funded by the Grain Research and Development Corporation (https://grdc.com.au). Grant number GRDC 9176106 to S.L.N and GRDC 9176093 to G.M.R. The publisher apologizes for the error.
