# Supplementary material for: Automated phenotyping for early vigour of field pea seedlings in controlled environment by colour imaging technology
Source: PLoS One. 2018 Nov 19;13(11):e0207788. doi: 10.1371/journal.pone.0207788 (PMC6242686; doi:10.1371/journal.pone.0207788)
Supplement: S1 Table — (DOCX) [file pone.0207788.s001.docx]

**S1 Table.** **Origin of 44 genetically diverse field pea varieties used in the study [52]**.

| **N^o^** | **Variety** | **Release year** | **Origin** | **Country** |
| --- | --- | --- | --- | --- |
| 1 | Alma | 1985 | SARDI, Australia | Australia |
| 2 | Bluey | 1992 | Agriculture Victoria, Australia | Australia |
| 3 | Bohatyr | 1996 | Czechoslovakia | Czechoslovakia |
| 4 | Bonzer | 1992 | Agriculture Victoria, Australia | Australia |
| 5 | Bundi | 2008 | Agriculture Victoria, Australia | Australia |
| 6 | Collegian | 1939 | SARDI, Australia | Australia |
| 7 | Cooke | 2000 | DAFWA, Australia | Australia |
| 8 | Cressy Blue | 1976 | Tasmania, Australia | Australia |
| 9 | Derrimut | 1964 | Agriculture Victoria, Australia | Australia |
| 10 | Dinkum | 1989 | Agriculture Victoria, Australia | Australia |
| 11 | Dunn | 1893 | England | England |
| 12 | Dundale | 1970 | SARDI, Australia | Australia |
| 13 | Dunwa | 2003 | DAFWA, Australia | Australia |
| 14 | Excell | 1999 | Agriculture Victoria, Australia | Australia |
| 15 | Glenroy | 1993 | SARDI, Australia | Australia |
| 16 | Helena | 2000 | DAFWA, Australia | Australia |
| 17 | Jupiter | 1993 | Cambrigde, England | England |
| 18 | Kaspa | 2002 | Agriculture Victoria, Australia | Australia |
| 19 | Kiley | 2002 | University of Sydney, Australia | Australia |
| 20 | King | 1998 | DAFWA, Australia | Australia |
| 21 | Laura | 1995 | SARDI, Australia | Australia |
| 22 | Magnet | 1998 | DAFWA, Australia | Australia |
| 23 | Maitland | 1986 | SARDI, Australia | Australia |
| 24 | Maki | 2011 | New Zealand/University of Sydney | New Zealand |
| 25 | Moonlight | 2005 | NSW DPI, Australia | Australia |
| 26 | Morgan | 1999 | NSW DPI, Australia | Australia |
| 27 | Mukta | 2000 | SARDI, Australia | Australia |
| 28 | Parafield | 2000 | SARDI, Australia | Australia |
| 29 | Paravic | 1999 | Agriculture Victoria, Australia | Australia |
| 30 | PBA Gunyah | 2010 | Agriculture Victoria, Australia | Australia |
| 31 | PBA Oura | 2010 | Agriculture Victoria, Australia | Australia |
| 32 | PBA Pearl | 2012 | Agriculture Victoria, Australia | Australia |
| 33 | PBA Percy | 2011 | Agriculture Victoria, Australia | Australia |
| 34 | PBA Twilight | 2010 | Agriculture Victoria, Australia | Australia |
| 35 | PBA Wharton | 2012 | Agriculture Victoria, Australia | Australia |
| 36 | Santi | 2000 | SARDI, Australia | Australia |
| 37 | Snowpeak | 2001 | Agriculture Victoria, Australia | Australia |
| 38 | Soupa | 2000 | SARDI, Australia | Australia |
| 39 | Sturt | 2005 | Agriculture Victoria, Australia | Australia |
| 40 | SW Celine | 2009 | Sweden | Sweden |
| 41 | Whero | 1979 | New Zealand | New Zealand |
| 42 | White Brunswick | 1920 | DAFWA, Australia | Australia |
| 43 | Wirrega | 1985 | SARDI, Australia | Australia |
| 44 | Yarrum | 2004 | New Zealand/University of Sydney | New Zealand |
